# Curcumin Attenuates Iron Accumulation and Oxidative Stress in the Liver and Spleen of Chronic Iron-Overloaded Rats

**DOI:** 10.1371/journal.pone.0134156

**Published:** 2015-07-31

**Authors:** Farid A. Badria, Ahmed S. Ibrahim, Adel F. Badria, Ahmed A. Elmarakby

**Affiliations:** 1 Department of Pharmacognosy, Faculty of Pharmacy, Mansoura University, Mansoura 35516, Egypt; 2 Department of Biochemistry and Clinical Biochemistry, Faculty of Pharmacy, Mansoura University, Mansoura 35516, Egypt; 3 Medical Technology Center, Alexandria University, Alexandria 21526, Egypt; 4 Department of Mechanical and Aeronautical Engineering, University of Patras, Patras, 26500, Greece; 5 Department of Oral Biology, Georgia Regents University, Augusta, Georgia, United States of America; University of Catania, ITALY

## Abstract

**Objectives:**

Iron overload is now recognized as a health problem in industrialized countries, as excessive iron is highly toxic for liver and spleen. The potential use of curcumin as an iron chelator has not been clearly identified experimentally in iron overload condition. Here, we evaluate the efficacy of curcumin to alleviate iron overload-induced hepatic and splenic abnormalities and to gain insight into the underlying mechanisms.

**Design and Methods:**

Three groups of male adult rats were treated as follows: control rats, rats treated with iron in a drinking water for 2 months followed by either vehicle or curcumin treatment for 2 more months. Thereafter, we studied the effects of curcumin on iron overload-induced lipid peroxidation and anti-oxidant depletion.

**Results:**

Treatment of iron-overloaded rats with curcumin resulted in marked decreases in iron accumulation within liver and spleen. Iron-overloaded rats had significant increases in malonyldialdehyde (MDA), a marker of lipid peroxidation and nitric oxide (NO) in liver and spleen when compared to control group. The effects of iron overload on lipid peroxidation and NO levels were significantly reduced by the intervention treatment with curcumin (P<0.05). Furthermore, the endogenous anti-oxidant activities/levels in liver and spleen were also significantly decreased in chronic iron overload and administration of curcumin restored the decrease in the hepatic and splenic antioxidant activities/levels.

**Conclusion:**

Our study suggests that curcumin may represent a new horizon in managing iron overload-induced toxicity as well as in pathological diseases characterized by hepatic iron accumulation such as thalassemia, sickle cell anemia, and myelodysplastic syndromes possibly via iron chelation, reduced oxidative stress derived lipid peroxidation and improving the body endogenous antioxidant defense mechanism.

## Introduction

Undiagnosed iron overload can lead to hemochromatosis, in which the excess iron stored in body organs is causing a serious tissue damage. Clinically, body iron level is primarily regulated by absorption rate as human subjects have no physiological mechanism by which excess iron is excreted. Accordingly, the level of dietary iron significantly influences iron absorption. Another important factor in regulating iron absorption relates to the form of iron present in a diet. Heme and non-heme iron are the two major sources of iron. Heme iron, mainly found in meat, fish, and poultry, is more effectively absorbed than non-heme iron due to its association with porphyrin ring [1]. A large population survey from Australia showed that average iron storage is about twice as much as the optimal iron store in normal adults. Heavy ethanol intake and high meat consumption are suggested to be the critical factors affecting iron absorption and storage in this population [2]. Additionally, iron overload is common in industrialized countries where red meat consumption and the use of iron fortification products are widespread [3]. Hepatotoxicity and spleen dysfunction are the most common pathological findings in patients with iron overload. The etiology of these multiple organ dysfunctions could be attributed to the presence of excess free iron released through the breakdown of heme by heme oxygenase (HO-1), which is ubiquitous abundant in such reticuloendothelial organs [4]. This free iron increases oxidative stress via generation of reactive oxygen species (ROS) [5] as well as depletes cellular stores of antioxidants [6]. Consequently, it is important to maintain iron homeostasis via ensuring proper iron supply while preventing accumulation of excess iron.

**Fig 1:**
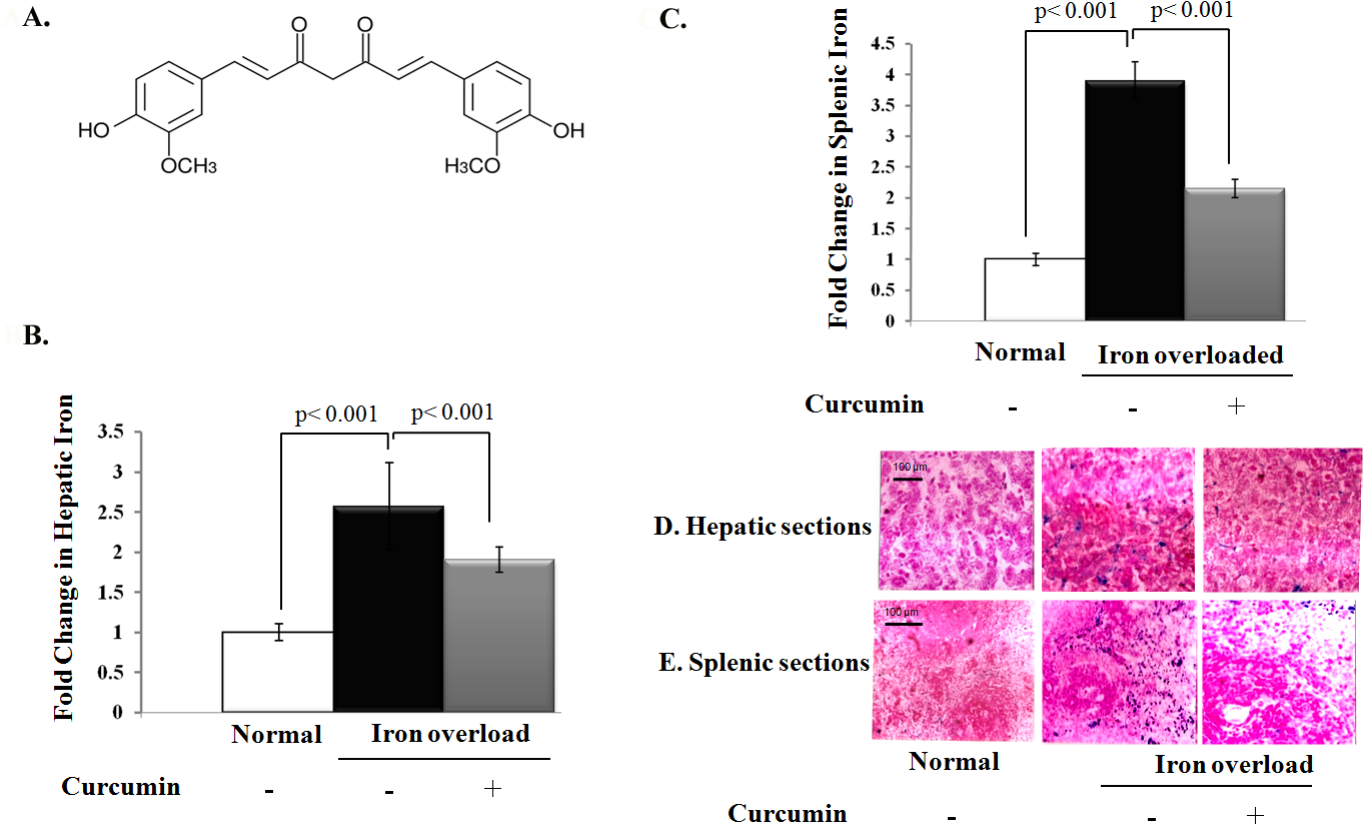
Potential effect of curcumin on iron accumulation within liver and spleen tissues. **A)** Structural characteristic of curcumin. **B, C)** Fold change in the level of iron (μmole/g tissue) in liver and spleen relative to that of normal control, which was assigned a value of 1. **D, E)** Histological examination of liver and spleen for iron overloaded rats with or without curcumin treatment using Prussian blue staining (blue stain).

In all iron overload-associated diseases such as thalassemia, sickle cell disease, and myelodysplastic syndromes, iron removal by iron chelation therapy is an effective and life-saving strategy. The current clinically available iron-chelating agents deferoxamine, deferiprone, and deferasirox show several side effects and limitations [7]. Accordingly, a new avenue is required to provide more effective treatment with lesser side effects to patients with iron overload. Curcumin, the main polyphenol in turmeric **(Fig 1A)**, has antioxidant, anti-inflammatory, and iron-chelating properties [8,9]. At molecular level, Curcumin downegulates various pro-inflammatory intracellular systems such as transcription factor nuclear factor κB (NFκB), inducible nitric oxide synthase, and hypoxia-inducible factor-1. Meanwhile, curcumin activates numerous antioxidant systems such as erythroid 2-related factor-2, and members of the vitagene family (e.g., heat shock protein 70, heme oxygenase-1 (HO-1), and thioredoxin) [10]. Given this complex array of interactions, curcumin represents a promising therapeutic option in the management of free radical-related diseases [11]. However, the effects of curcumin on hepatic and splenic abnormalities induced by iron overload have not been clearly investigated.

Previously, we reported the deleterious impact of excessive environmental iron on human health among Egyptians who live in an area with high iron content in drinking water [12,13]. In the current study, we aim to determine whether curcumin supplementation would attenuate hepatic and splenic abnormalities induced by iron overload and to explore the underlying mechanisms.

## Materials and Methods

### Materials

Na_2_HPO_4_, KH_2_PO_4_, p-phenylenediamine.HCl, and NaNO_2_ were purchased from Merck (Darmstadt, Germany). Nitrobluetetrazolium (NBT), Potassium Ferrocyanide, phenazinemethosulfate (PMS), NADH, 5,5-dithiobis (2-nitrobenzoic acid) (DTNB), thiobarbituric acid (TBA), sodium dodecyl sulfate (SDS), curcumin, and dianisidine dihydrochloride were obtained from Sigma (St Louis, MO, USA).

### Animal care and treatments

All procedures with animals were performed in accordance with the National Institutes of Health Guide for the Care and Use of Laboratory Animals and approved by the Research Ethics Committee at the Faculty of Pharmacy, Mansoura University (Mansoura, Egypt). Young albino male Sprague Dawley rats were obtained from Theodor Bilharz institute, Imbaba, Cairo, Egypt at 6–8 weeks old with an average body weight of 150 gm. Rats were housed in well-ventilated opaque polypropylene cages. All animals had a free access to balanced laboratory diet and water ad libitum. Water is municipality tap in drinking bottles. Animals were acclimated to the housing conditions (12-h light/dark cycle, temperature 25°C, relative humidity (40%–60%) for at least 5 days prior to initiation of experiment and were then divided randomly into three groups (n = 5); group 1 (control rats without iron supplementation), group 2 (rats supplemented with iron in a drinking water for 60 days and then left untreated for 60 more days) and group 3 (rats supplemented with iron in a drinking water for 60 days then treated with 100 mg/kg/day curcumin suspended in drinking water for 60 more days). The concentration of iron used was calculated to exceed the maximum permissible concentration (MPC) for this chemical in Egyptian Ministry of Health. MPC for Fe^2+^ is 0.3 mg per liter of drinking water. Control rat group (group 1) received tap water whereas iron overloaded groups (group 2 & 3) received drinking water containing 3 mg/L of Fe^2+^ (using 8.3 mg/L of FeSO4). Water consumption was recorded on daily bases and the dose of curcumin was selected based on previous studies [14]. In most animal studies, a dose range of 50–200 mg/kg body weight curcumin exhibited a good anti-inflammatory activity and seemed to have negligible adverse effect on human [15].

### Samples Collection

At the end of the experiment, rats were anesthetized with isoflurane and killed by decapitation. Liver and spleen tissues were excised and divided into 2 portions. One portion immediately was frozen at −80°C for biochemical analysis. The other portion was fixed in 10% neutral buffered formalin for histological examination.

### Preparation of homogenates

One gram of liver and spleen from each rat was homogenized in 10 mL ice cold homogenate buffer (0.3 M sucrose and phosphate buffer; pH 7.4) using a Teflon pestle connected to a Braun Homogenizer Motor (25 strokes/min at 1000 rpm). The homogenate was centrifuged at 30,000 x g for 30 min at 4°C to remove cell debris and nuclei. The resulting supernatant was used for biochemical analysis.

### Estimation of iron and copper levels in liver and spleen tissues

Briefly, liver or spleen homogenates were digested with HNO_3_ and the residues were dissolved in 0.1 mol/L HNO_3_. Samples were then analyzed for copper and iron by flame atomic absorption spectroscopy (Perkin-Elmer 2380, Norwalk, CT 06859–0012, USA) using an air acetylene flame.

### Determination of Lipid Peroxidation as an indicative of oxidative stress

The amount of lipid peroxidation was determined in term of malonyldialdehyde (MDA). Briefly, 0.8% TBA (1.5 ml), 8.1% SDS (200 μl), 20% acetic acid (1.5 ml) and distilled water (600 μl) were added to 200 μl tissue homogenate at temperature of 95°C for 30 min and immediately cooled on ice to form colored product. The resultant pink color was a representative of thiobarbituric acid-reactive substances (TBARS) and was measured colorimetrically at 534 nm using a spectrophotometer [16,17].

### Measurement of NO concentration in liver and spleen tissues

Liver and spleen nitrite (NO_2_^-^) concentrations, a stable metabolic product of NO with oxygen were assessed as indirect indicative of tissue NO levels. Briefly, conversion of nitrate (NO_3_^-^) into NO_2_^-^ was carried out in the presence of elementary zinc. NO_2_^-^ concentration in tissues was determined by the classic colorimetric Griess reaction. Briefly, equal volumes of tissue homogenate and Griess reagent were mixed at room temperature and the absorbance was measured colorimetrically at 570 nm using a spectrophotometer. The concentration of NO_2_^-^ was determined using sodium nitrite standard curve [18].

### Determination of glutathione and ascorbic acid, in liver and spleen tissues

Levels of reduced glutathione in liver and spleen homogenates were assessed by adding DTNB to form a stable yellow colored complex that was measured spectrophotometrically at 412 nm [19]. Levels of ascorbic acid in tissue homogenates were also assessed by its oxidation using Cu^+2^ to form dihydroascorbic acid, which reacted with acidic 4- dinitrophenyl hydrazine to form a red hydrazones. The resultant red color was measured spectrophotometrically at 520 nm [20].

### Determination of antioxidant enzymes, Superoxide-dismutase (SOD) and catalase (CAT)

Tissue homogenates were used for measuring the activity of the antioxidant enzymes, SOD and CAT, using standard spectrophotometric assays. Briefly, SOD activity in the tissue homogenates was determined by generating superoxide radicals using photochemical reduction of phenazine methosulphate, which reduces nitrobluetetrazolium into a blue-colored compound, formazone. SOD quenches free oxygen radicals and inhibits reduction of nitroblue tetrazolium, which was measured colorimetrically at 560 nm [21]. CAT assay was carried out by assessing the rate of hydrogen peroxide degradation at 510 nm in the presence of tissue homogenate [22].

### Determination of ceruloplasmin activity in liver and spleen tissues

Ceruloplasmin activity was determined by measuring the ability of tissue homogenates to oxidize o-dianisidine [23]. The assays were conducted using 0.1 mol/L sodium acetate buffer. The reaction was initiated by adding 30 ul of dianisidine dihydrochloride to small test tubes containing 112.5 ul of buffer and 7.5 ul of tissue homogenate. The absorbance of the acidified product was measured colorimetrically at 540 nm over 30 minutes to determine ceruloplasmin activity.

### Histological Analysis

Hepatic and splenic tissues were excised, and then fixed in 10% neutral buffered formalin. Tissues were then processed for paraffin embedding and were subsequently sectioned at 3–4μm (Reichert Jung microtome, Germany). Deparaffinized sections were stained with hematoxylin/eosin (H&E) and Prussian blue stains to assess iron distribution [24].

### Statistical analysis

All analyses were performed with the Graph Pad Prism 3 software. Data are presented as means ±SD. Statistical differences were evaluated by one-way analysis of variance (ANOVA) followed by checking for skewness. An unpaired t-test was performed if the distribution of the values was Gaussian. If the distribution was not normal, a Mann-Whitney test was used. P values less than 0.05 were considered statistically significant.

## Results

### Increased iron intake increased iron density and induced oxidative stress in liver and spleen and these changes were reduced by curcumin treatment

Initially, there were no significant changes in water and food consumption or behavior among rat groups during iron overload. We then tested whether curcumin decreases the iron accumulation in liver and spleen of iron overloaded rats. As shown in **Fig 1B and 1C,** chronic iron supplementation led to about 2.6- and 3.9-fold elevation in total hepatic and splenic iron content, respectively, when compared to the corresponding values of control rats. Curcumin resulted in a significant reduction in iron content in liver and spleen of iron overloaded rats (*P* < 0.001). We also investigated the distribution of iron in liver and spleen of iron overloaded rats using Prussian blue staining. The results of Prussian blue staining showed that the hepatic cells and splenic parenchymatic cells have the greatest iron density. Curcumin treatment also significantly reduced the increase in iron density in the hepatic cells and splenic parenchymatic cells of iron overloaded rats (**Fig 1D and 1E**).

**Fig 2:**
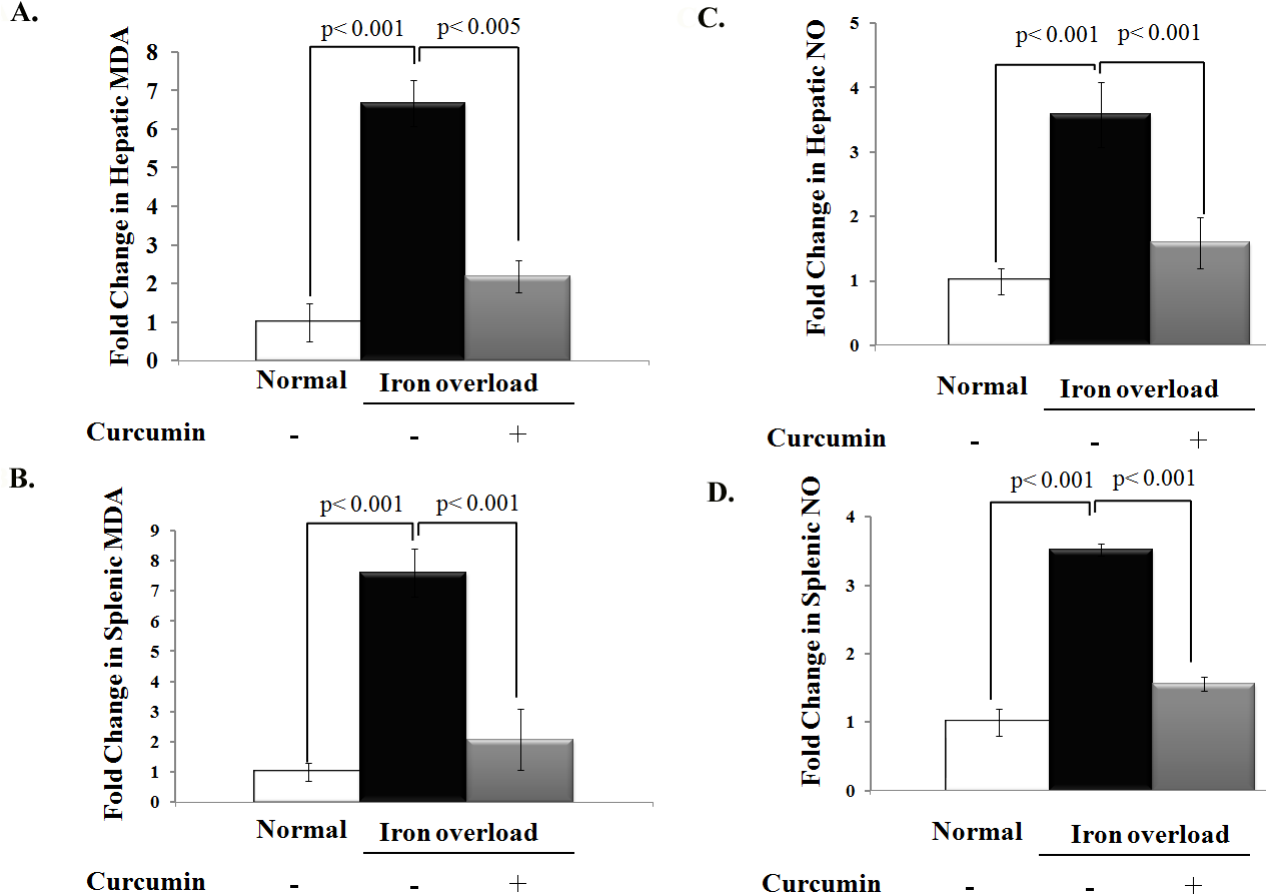
Effect of iron overloading on oxidative stress and NO levels in liver and spleen and potential protective effect of curcumin. **A, B)** Fold change in the level of malonyldialdehyde (MDA) as a marker of oxidative stress in liver and spleen (nmole/ g wet tissue), respectively, relative to that of normal control, which was assigned a value of 1. Levels of MDA in liver and spleen were significantly increased in iron overloaded rats relative to normal controls and curcumin treatment significantly decreased MDA levels in these tissues during iron overload. **C, D)** Fold change in the level of NO (nmole/ g wet tissue) in liver and spleen tissues, respectively, relative to that of normal control. Levels of NO in these tissues were significantly increased in iron overloaded rats than in control rats and was significantly reduced by curcumin treatment in iron overloaded rats. Data shown are the mean ± SD (n = 5).

Next we examined the effect of curcumin on iron overload–induced malonyldialdehyde (MDA) production, a terminal compound of lipid peroxidation that is commonly used as an index of oxidative stress. Chronic iron overload resulted in a significant increase in hepatic and splenic MDA levels and curcumin treatment normalized the elevation in MDA in iron overloaded rats (P< 0.05, **Fig 2A and 2B**). Additionally, the increase in oxidative stress in iron overloaded rats was associated with a marked increase in liver and spleen NO levels (P< 0.05). Curcumin supplementation significantly reduced the elevation in hepatic and splenic NO levels in iron overloaded rats as shown in **Fig 2C and 2D**.

### Curcumin boosts levels of endogenous antioxidants that increased iron-intake depletes

**Fig 3:**
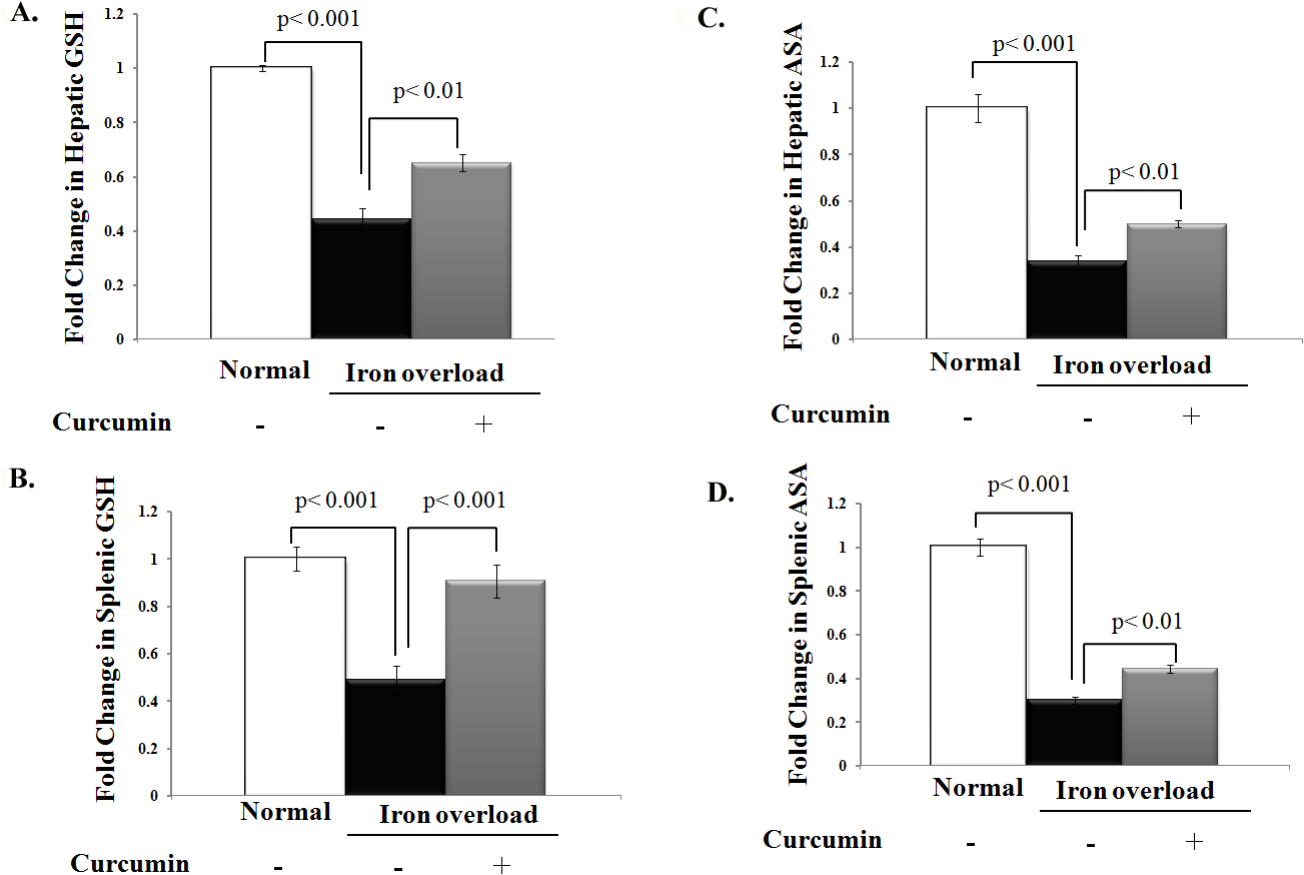
Curcumin improves levels of endogenous non-enzymatic antioxidants that were depleted by chronic iron intake. **A, B)** Fold change in the level of reduced glutathione (GSH, mg/g wet tissue) in liver and spleen, respectively, relative to that of normal control. **C, D)** Fold change in the level of ascorbic acid (ASA, mg/g wet tissue) in liver and spleen, respectively, relative to that of control. Levels of GSH and ASA in liver and spleen were decreased in iron-overloaded rats and curcumin treatment significantly improved the decrease in GSH and ASA levels in iron overloaded rats. Data shown are the mean±SD (n = 5).

We next determined whether curcumin alleviation of the iron overload-induced oxidative stress is also attributed to the restoration of endogenous antioxidant defense system. As shown in **Fig 3A and 3B**, chronic iron intake was clearly associated with a significant depletion in hepatic and splenic reduced glutathione when compared to control rats (P< 0.05). Curcumin treatment resulted in a significant improvement in liver and spleen reduced glutathione levels by approximately 65% and 90%, respectively, in iron overloaded rats. Similarly, ascorbic acid levels in the liver and spleen of iron-overloaded rats were 60–70% lesser than control rats (P< 0.05) and curcumin treatment increased liver and spleen ascorbic acid levels in iron overloaded rats significantly (**Fig 3C and 3D**).

**Fig 4:**
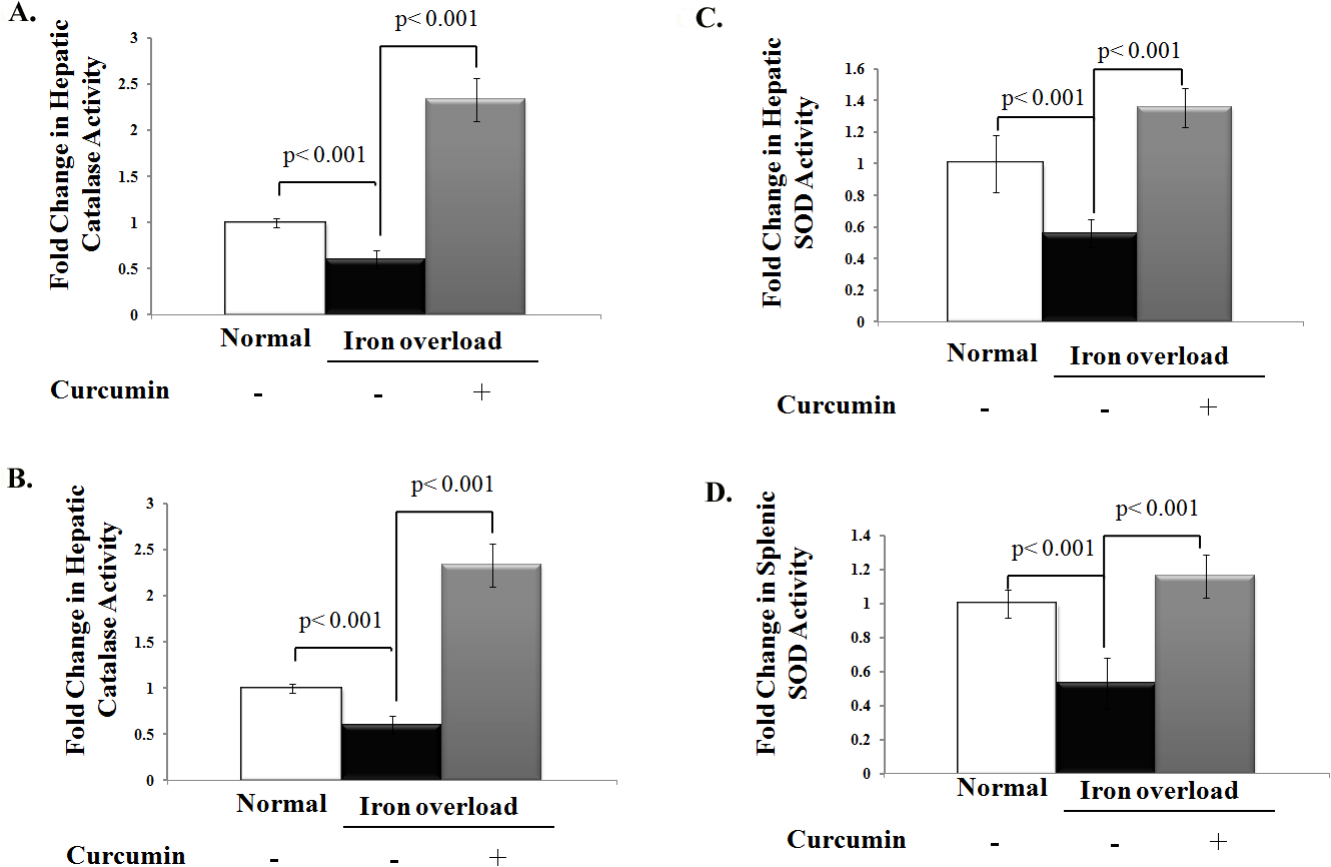
Curcumin boosts activities of endogenous enzymatic antioxidants that were depleted with chronic iron overload. **A, B)** Fold change in catalase (CAT) activity (U/g tissue) in liver and spleen relative to that of normal control, which was assigned a value of 1; **C, D)** Fold change in Superoxide dismutase (SOD) activity (U/g tissue) in liver and spleen relative to that of normal control which was assigned a value of 1. Activities of CAT and SOD in these tissues were significantly decreased in iron overloaded rats than controls. Curcumin treatment significantly boosted activities of both CAT and SOD in iron overloaded rats even to higher levels than those of control rats. Data shown are the mean±SD (n = 5).

**Fig 5:**
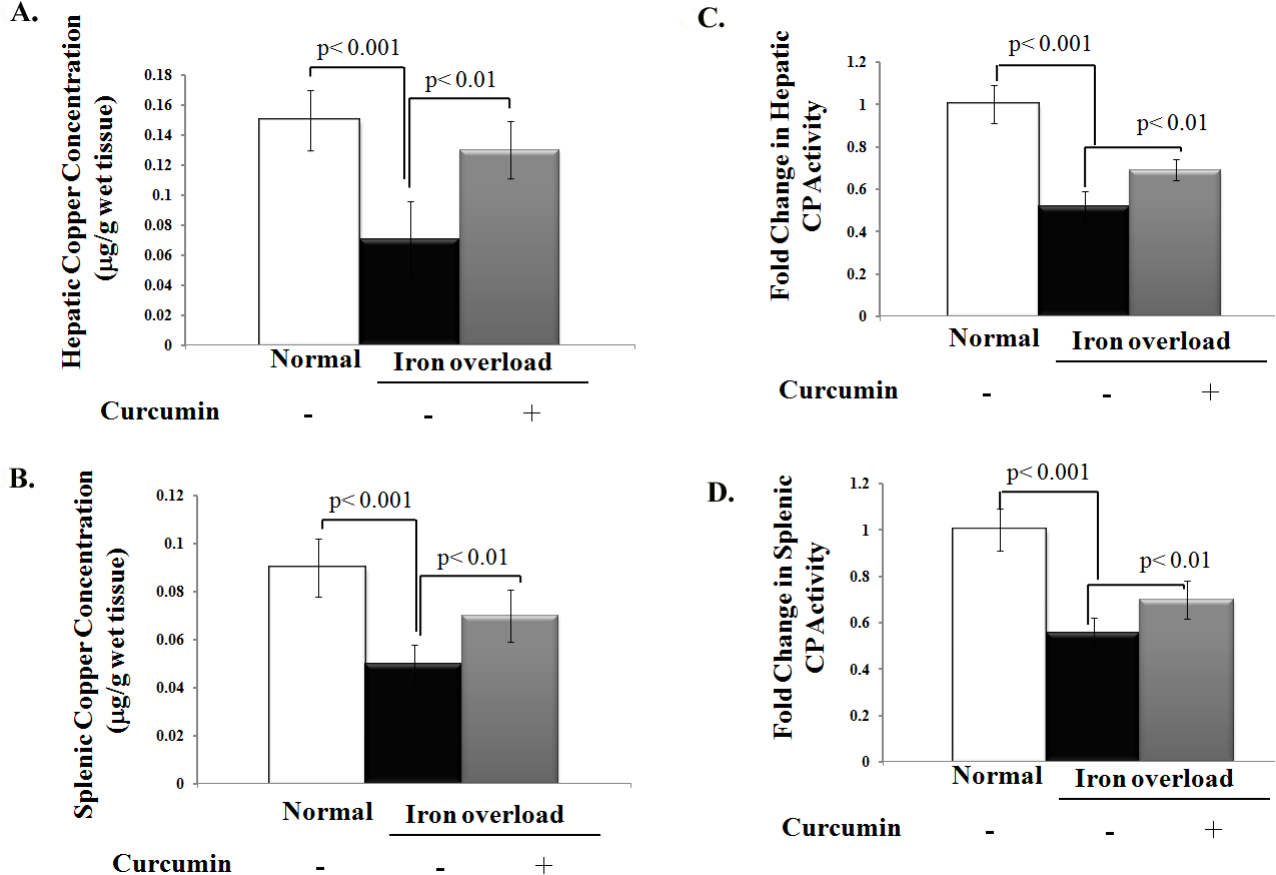
Curcumin’s modulation of copper content and antioxidant protein (ceruloplasmin) activity in liver and spleen of iron overloaded rats (A, B). Levels of copper in both liver and spleen homogenates of iron overloaded rats treated with or without curcumin; (**C, D)** ceruloplasmin activity (U/g tissue) in liver and spleen relative to normal control, which was assigned a value of 1. Compared to the corresponding values of the control rat group, the administration of curcumin in iron overloaded rats significantly enhanced the decrease in copper levels and ceruloplasmin activity in both liver and spleen homogenates. Data shown are the mean±SD (n = 5).

Chronic iron overload also lowered catalase activity in both liver and spleen homogenates by 40 and 50%, respectively, when compared to control group. However, curcumin treatment restored iron overload-induced depletion of catalase activity to levels even higher than control group (**Fig 4A and 4B**). Similarly, curcumin treatment significantly boosted the decreased in hepatic and splenic Cu- SOD activity in iron overloaded rats by approximately 2.4- and 2.2-fold, respectively, (**Fig 4C and 4D**). Because copper deficiency could be the common cause for decreased SOD activity in iron overloaded rats and the enhancement of liver and splenic SOD activity upon curcumin treatment could be attributed to the modulation of organ copper content and its antioxidant protein ceruloplasmin, we finally assessed copper level and ceruloplasmin activity in iron overloaded rats with or without curcumin treatment as shown in **Fig 5.** Both cupper level and ceruloplasmin activity significantly decreased in the liver and spleen homogenates of iron overloaded rats compared to control group (**Fig 5A and 5B).** Curcumin treatment increased copper levels and ceruloplasmin activity in liver and spleen homogenates of iron overloaded rats (**Fig 5C and 5D)**; however, these changes remained lesser than control group.

## Discussion

The current study demonstrated that chronic iron intake in rats increases hepatic and splenic iron contents and these changes were associated with elevation in lipid peroxidation and decreased antioxidant defense mechanisms. Curcumin supplementation decreases hepatic and splenic iron density as well as oxidative stress produced by chronic iron intake together with an improvement in antioxidant defense mechanism. These data suggest that curcumin supplementation could be a potential new therapeutic option to combat pathological diseases associated with iron accumulation.

Iron overload could result from increased dietary iron absorption, hereditary hemochromatosis, chronic liver diseases, and diseases associated with hemolytic anemia such as β-thalassemia [25]. We have previously reported the impact of excess iron on human health in the area with high iron content in drinking water [12,13]. Current treatment of iron overload includes iron chelators, which are hampered by their off-target and side effects. For example deferoxamine is a commonly used iron chelator; however, it has limited capacity to enter cells and is rapidly metabolized [26,27,28]. Other iron chelators, such as deferiprone, increases risk of agranulocytosis and neutropenia [29], whereas deferasirox increases risk of gastrointestinal disturbances and hepatic failure [30]. These therapeutic limitations of current available iron chelators highlight the need for alternative pharmacological interventions. In the current study, we provide a pre-clinical evidence to use curcumin, a micronutrient with multi-target therapeutic effects, to treat complications associated with increased iron accumulation. Curcumin possesses anti-inflammatory properties when compared to the currently used iron chelators [13] and its efficacy has been demonstrated in different animal models for diseases associated with oxidative injury [31,32].

It has been shown that excess iron content is responsible for functional abnormalities during chronic iron overload; therefore, curcumin supplementation might provide beneficial effects during chronic iron overload via chelating with free iron [33]. The specific metal chelator effect of curcumin has been established previously in cell free system and was attributed to the presence of chemical groups such as β-diketonate group [34]. This assumption was supported using spectrophotometric quantification of curcumin affinity to copper, zinc, and iron ions. Although Zn^2+^ showed little binding affinity to curcumin, Cu^2+^ and Fe^2+^ appeared to bind at least two curcumin molecules [35]. *In vivo* studies demonstrated that curcumin induced iron depletion in mice with low levels of body iron [36]. Consistence with these findings, our study suggests that curcumin decreased iron levels in the liver and spleen of chronic iron overloaded rats possibly via iron chelation.

Excess iron content in the cell is potentially detrimental because it is involved in oxidation-reduction reactions, which in turn promote tissue injury by catalyzing lipid peroxidation [6]. Iron initiates lipid peroxidation by producing highly reactive hydroxyl radicals from hydrogen peroxide via Fenton type reactions or by complexing with oxygen directly to yield reactive perferryl and ferryl ions [5]. The role of oxidative stress in the pathogenesis and progression of other diseases, including cardiovascular disorders, is well established. Additionally, curcumin has been shown to reduce oxidative stress in cardiovascular diseases [37,38]. This raises the possibility that curcumin could provide beneficial antioxidant effects during chronic iron overload beyond its role as an iron-chelating agent, such as the possession of antioxidant property. The antioxidant property of curcumin is endorsed by the presence of chemical groups such as hydroxyl, methoxy and 1,3-diketone conjugated diene system in curcumin structure [39]. Previous studies have shown that curcumin significantly reduced the redox activity of iron and lowered the elevation in liver and serum lipid peroxide levels during iron injection [40]. Curcumin also reduced iron overload-induced reactive oxygen species generation and subsequent activation of NF-κB, the key regulatory transcription factor for the inflammation-related gene expression, in cultured hepatocytes [41]. Our study supports previous findings as curcumin supplementation reduced lipid peroxidation during chronic iron overload. The increased in lipid peroxidation during chronic iron overload was associated with elevation in hepatic and splenic NO levels which could be a compensatory mechanism to quench the elevation in oxidative stress. This assumption is supported by the fact that curcumin-induced lowering in lipid peroxidation was also associated with decrease in liver and spleen NO levels during iron overload.

The overall reduction in oxidative stress by curcumin treatment could be also attributed to its ability to restore activities of endogenous antioxidant defense mechanism as shown previously [42]. In our study, the elevation in oxidative stress in chronic overloaded-rats was associated with depletion of putative non-enzymatic (reduced glutathione and ascorbic acid) as well as enzymatic (catalase, superoxide dismutase and ceroplusmin) antioxidants in liver and spleen. Furthermore, curcumin supplementation significantly restored the depletion levels of GSH and ASA as well as the activities of enzymatic antioxidants. Although catalase is an iron-dependent enzyme, significant decrease in catalase activity has been shown in the current study, similar to what previously reported in iron overload toxicity [43]. This discrepancy between iron content and decrease in calatase activity could be attributed to the destruction of heme by iron-induced peroxidation [44]. The decrease in catalase activity could also be attributed to decreased cupper content because curcumin-induced increases in hepatic and splenic cupper content was associated with elevation in catalase activity during chronic iron overload. Since the depletion of the antioxidant defense was significantly restored by curcumin treatment, our study suggests that curcumin treatment could alleviate the increase of hepatic and splenic oxidative stress during increased iron intake both directly via decreased lipid peroxidation and indirectly via restoring the levels of the depleted endogenous antioxidant defense mechanism.

Besides curcumin’s ability to restore activities of GSH, ASA, CAT, and SOD, other reported effects might have contributed to our finding of curcumin-anti-oxidant effect, such as the induction of HO-1, a redox-sensitive inducible protein that provides protection against various forms of stress [10]. The reaction products of HO-1 induction, biliverdin, and its subsequent metabolite, bilirubin, have antioxidant properties [45]. However, iron released from HO-1 induction can convert H_2_O_2_ to the highly reactive OH• [4]. At first glance, up-regulation of HO-1 by curcumin would seem to be counterintuitive, since uncontrolled release of iron into a cell would promote the Fenton reaction. However, in conjunction with HO-1 up-regulation, curcumin detoxifies iron by subsequently sequestrating it within its own structure where it cannot be utilized for the Fenton reaction [35,36].

These multiple effects of curcumin envision its future clinical use as a promising treatment for iron overload. However, some adverse effects may arise during curcumin treatment, such as stomach upset as well as impaired activity of hepatic drug-metabolizing enzymes, including cytochrome P450, glutathione-S-transferase, and UDP-glucuronosyltransferase, leading to increased toxicity of co-administered drugs [46,47,48]. These raise clinical concerns for using curcumin in patients with gastroesophageal reflux disease or those taking drugs metabolized by the aforementioned enzymes, such as digoxin, acetaminophen and/or morphine. Moreover, the lack of long-term toxicity studies in humans and limited curcumin bioavailability still represent potential barriers against its clinical utilization [47,49,50]. Nevertheless, future direction will focus on improving curcumin bioavailability while using an appropriate dose of curcumin that will not significantly disrupt iron homeostasis. This will likely bring curcumin as a valuable therapeutic option for patients at high risk of chronic iron accumulation.
