# Correction: Curcumin attenuates iron accumulation and oxidative stress in the liver and spleen of chronic iron-overloaded rats

**DOI:** 10.1371/journal.pone.0243398

**Published:** 2020-12-01

**Authors:** Farid A. Badria, Ahmed S. Ibrahim, Adel F. Badria, Ahmed A. Elmarakby

The image for Fig 4 is incorrect. Please see the correct Fig 4 here.

**Fig 4:**
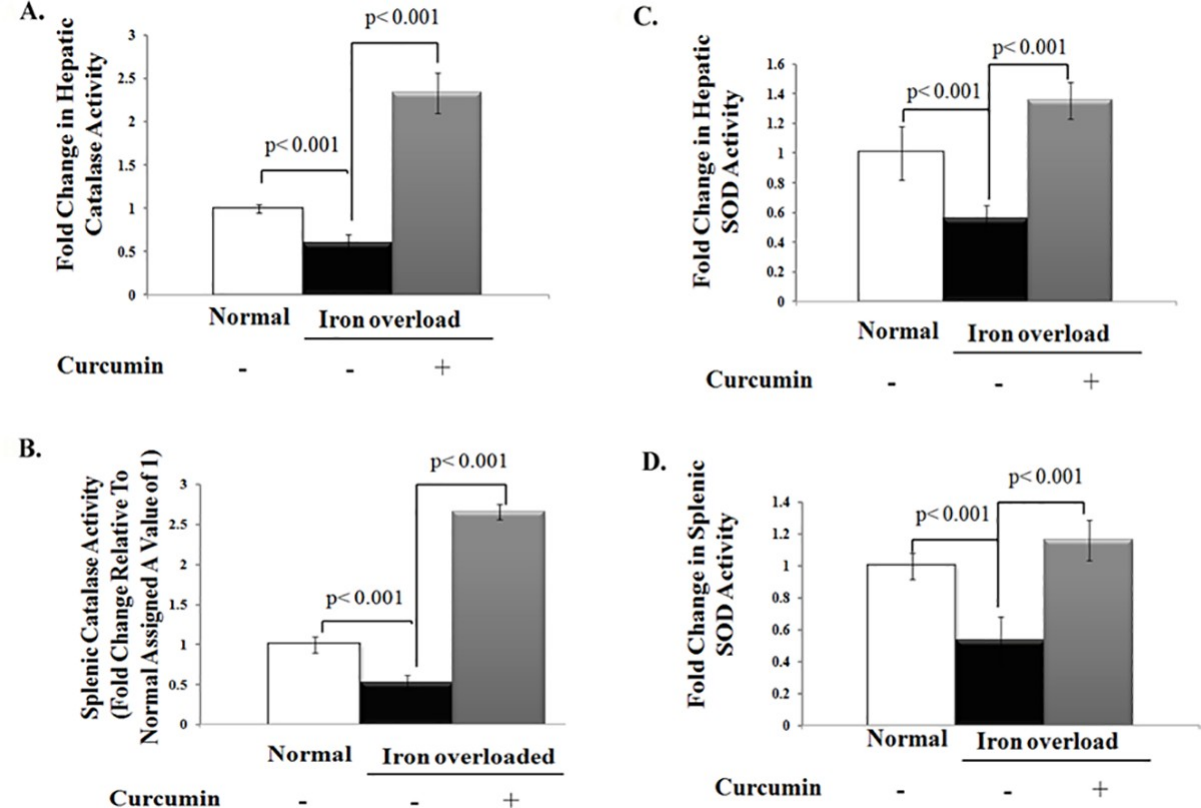
Curcumin boosts activities of endogenous enzymatic antioxidants that were depleted with chronic iron overload. **A, B)** Fold change in catalase (CAT) activity (U/g tissue) in liver and spleen relative to that of normal control, which was assigned a value of 1; **C, D)** Fold change in Superoxide dismutase (SOD) activity (U/g tissue) in liver and spleen relative to that of normal control which was assigned a value of 1. Activities of CAT and SOD in these tissues were significantly decreased in iron overloaded rats than controls. Curcumin treatment significantly boosted activities of both CAT and SOD in iron overloaded rats even to higher levels than those of control rats. Data shown are the mean±SD (n = 5).
