# Correction: Curcumin attenuates iron accumulation and oxidative stress in the liver and spleen of chronic iron-overloaded rats

**DOI:** 10.1371/journal.pone.0321563

**Published:** 2025-04-02

**Authors:** Farid A. Badria, Ahmed S. Ibrahim, Adel F. Badria, Ahmed A. Elmarakby

An additional affiliation is missing for the fourth author. Ahmed A. Elmarakby is also affiliated with the Department of Pharmacology & Toxicology, Faculty of Pharmacy, Mansoura University, Mansoura 35516, Egypt.
